# School principals’ span of control and its cross-sectional associations with signs of exhaustion, perceived work ability, and demanding and supportive circumstances in managerial work

**DOI:** 10.1186/s12889-026-27265-9

**Published:** 2026-04-21

**Authors:** Roger Persson, Ulf Leo, Anna Oudin, Carita Håkansson, Kai Österberg

**Affiliations:** 1 Department of Psychology, Lund University, Lund, SE-22100 Sweden; 2 Centre for Principal Development, Umeå University, Umeå, Sweden; 3 Division of Occupational and Environmental Medicine, Lund University, Lund, Sweden

**Keywords:** Compulsory school, Education, Exhaustion, LUCIE, Pre-school, Self-rated health, Stress, Upper secondary school, WAS, Wellbeing

## Abstract

**Background:**

To educate students and prepare them for life in society, school principals, teachers, and staff need proper work-related organizational preconditions, motivation, and good health. In this context, span of control (SoC, i.e., the number of employees a manager is accountable for) is an understudied modifiable organizational precondition that influences the relationships between managers and employees. Therefore, as a basis for future preventive actions, we examined (a) school principals’ SoC and (b) how SoC varied across gender, job title, years of working as a school principal, and school owner, and the extent to which SoC was associated with (c) the reporting of exhaustion symptoms and perceived work ability, and (d) demanding and supportive managerial circumstances.

**Methods:**

School principals (*N* = 2045; mean age 49 years [SD 7 years]; 77% women) in Sweden completed a cross-sectional web survey including background information and the Lund University Checklist for Incipient Exhaustion, a brief version of the Gothenburg Manager Stress Inventory, and the Work Ability Score. SoC was assessed as the total number of employees the principal was accountable for. Median and quartile splits created four groups with incrementally wider SoC. Data were analysed using non-parametric tests as well as multivariate analyses of variance (MANOVAs).

**Results:**

The mean SoC was 31.7 employees (SD 15.5). SoC differed across job titles, school levels, school owner, and years of working as a school principal. There was no difference in SoC across gender, exhaustion symptoms, or work ability. Adjusted MANOVAs and subsequent post-hoc testing indicated that principals with the narrowest SoC (i.e., ≤ 22 employees) reported less frequent role conflicts and role demands, and less frequent need to harbour employee’s frustration as well as less access to support from colleagues at the same level.

**Conclusions:**

Irrespective of school ownership, an SoC of no more than 22 employees was consistently associated with reports of perceiving less frequent role conflicts, role demands, and need to harbour employee frustrations. Presumably, reducing school principals’ SoC to 22 employees or fewer may alleviate the burden caused by role conflicts and demands, though it may not directly impact exhaustion, work ability, or access to supportive colleagues.

## Background

Like in many other countries, during recent decades the educational system in Sweden has been shaped by ideas from New Public Management (NPM) [1]. This has led to an intensification of work and an increased emphasis on accountability, decentralization, marketization, and management by objectives and results, which has led many school principals to adopt a work role similar to that of private-sector-style managers [2]. According to the Teaching and Learning International Survey (TALIS) [3], Swedish school principals primarily report being stressed by having too much administrative work, having extra duties due to absent school staff, and having to accommodate students with special needs [4]. In addition, circa 20% to 25% of Swedish school principals express a symptomatology with signs of exhaustion that, if sustained, might lead to poor health [5], and they rate their work ability slightly lower compared with a Swedish stratified random national sample [6, 7]. Recent research [8–10] and official reports [11, 12] has also shown that some schools struggle with the consequences following high and unwanted principal turnover (e.g., lack of continuity in leadership, increased costs and administration). All things considered, the situation would seem to call for better work organization, as the co-operative efforts of school principals, teachers, and other allied staff at the local school unit are key to completing the mission of educating children and students and preparing them for life in society [13, 14].

As managers, school principals are responsible for the school’s internal organization, pedagogical development, and the work environment of teachers and students [15]. The number of employees they are accountable for defines their span of control (SoC, also known as span of management, span of authority, span of command, or span of responsibility) [16]. Noticeably, the number of employees a school principal is formally accountable for does not necessarily mirror the number of employees the school principal regularly interacts with, nor does it reveal the nature and quality of interactions. However, because SoC is a modifiable organizational precondition that influences the relationship between school principals and employees, SoC stands out as a potential intervention area. A broad SoC (i.e., being accountable for many employees) limits the time a school principal can engage with individual employees and is likely to affect the time allocation for other work tasks as well. Yet, in light of relationship-based leadership theory, which underline that both the nature and quality of interactions between managers and employees are central to achieve effective management and employee well-being [17], it is conceivable that a broader SoC under certain circumstances may be beneficial even if it inevitably limits the managers time to engage with each individual employee. For example, increasing the number of qualified teachers could, in theory, improve the quality of supervision and alleviate the school principals’ workload even if the time available for interacting with each individual teacher will be reduced.

That said, SoC is at its core a quantitative concept and theory stipulates a curvilinear relationship with performance outcomes. As regards achieving effective supervision, it is assumed that SoC can be both too narrow and too broad [16]. For example, a recent Danish study showed that, in a hospital setting, a medium SoC (i.e., 30 to 40 individuals) was associated with greater job satisfaction compared with both narrower (i.e., 6 to 29 individuals) and broader SoCs (i.e., 40 to 118 individuals) [18]. In contrast, but also in a hospital setting, Cathcart and colleagues reported that employee engagement noticeably dropped when the group size grew larger than 15 individuals per manager and then dropped again at 40 individuals per manager, suggesting the possibility of “critical points” [19]. However, research has yet to identify a gold standard for “critical points” or for “narrow”, “medium” and broad” SoCs. Past suggestions range from no limit, to specific numbers (e.g., 5, 10, 15, 50 to 60) or ratios expressing a proportionality [16]. Then again, it is believed that there is a limit that defines the possibilities for effective management, but also that this limit will vary depending on the individual manager, the group members, and the situation [16] or on other factors such as tradition, environmental uncertainty, level of technology, subordinates’ task characteristics, leadership behaviour, and the leader’s workload [20]. Moreover, diversification of function, stability of resources and staff, size, and geography have also been suggested as determinants of SoC [21]. Collectively, these observations suggest that any research studying the effects of SoC needs to be contextualized as well as that the findings will have limited generalizability beyond the selected context and/or situation.

Previous research on SoC has been described as rather sparse and often lacking in solid empirical foundations [21] and as not paying attention to how SoC can influence managers and their preconditions for work [22]. Moreover, the need to further our general knowledge about leaders’ preconditions, specifically SoC, has been acknowledged [23]. The notion that SoC may have implications for managers’ working conditions is conceivable in light of a Swedish study that examined the concept in relation to seven perceived job demands (i.e., work overload, conflicting logics, excessive role demands, buffer problems, container function, group problems, and individual employee problems) [22]. While studying an assortment of 434 public sector managers within human services, care of the disabled and elderly, pre-school and compulsory school, upper secondary school, and technical services, it was observed that the mean SoC was 27 subordinates (range 2 to 150). It was also concluded that, for most public sector managers, having many subordinates was disadvantageous, and of the seven job demands examined, excessive role demands and demands of dealing with co-workers’ frustrations and problems (i.e., a container function) were the most strongly associated with SoC [22]. At any rate, because school principals, like other managers, need proper organizational preconditions, motivation, and good health to do their job, increased knowledge about SoC and its potential associations with indicators of mental health, work ability, and managerial circumstances may shed light on the potential value of SoC interventions and may serve to inform and guide future preventive actions.

### Objectives

The present study had four aims. The first aim was to map and describe the SoC of Swedish principals working in public and independent pre-schools, compulsory schools, upper secondary school, and adult education. The second aim was to examine to what extent SoC varied across demographic and organizational factors such as gender, job title, school owner and experience of working as a school principal. The third aim was to examine to what extent groups of school principals with varying spans of control differed in their reporting of signs of exhaustion as well as perceived work ability. The fourth and final aim was to examine to what extent groups of school principals with varying spans of control differed in their reports of demanding and supportive circumstances in their executive work. Presumably, attempting to answer these questions will generate knowledge on Swedish principals’ SoC that can inform stakeholder groups on the national, municipal, and local level (including the principals themselves) about the potential benefits to health, work ability, and managerial circumstances that may be associated with narrower and broader spans of control.

## Methods

### Study design

The present study is part of a larger project on Swedish school principals that was designed to examine multiple questions, some of which have already been addressed in previous work [5, 7, 24, 25]. The present cross-sectional study draws on data collected using the software Textalk Websurvey (www.textalk.se; Gothenburg, Sweden) between September 25 and October 23, 2018. Four reminders were sent out (first: October 1; last: October 21). The response window was selected to allow assessment during a period with a relatively stable workload in a work year that comprises both peak loads and holidays. To ensure quality, the participants were told to read the questions carefully, reserve 30 min to complete the survey, and that survey responses could be stored temporarily if they were short on time.

### Study setting: the Swedish educational system

In Sweden, the educational system is governed by the state via statues, rules, national curriculums, and syllabuses, whereas the 290 municipalities, the independent school owners, and the local school units are responsible for implementing the state directives [15]. Organizationally, the educational system comprises various school levels (e.g., pre-school, compulsory school including pre-school class, upper secondary school, adult education, special schools), and both public and private actors are allowed to own and operate schools in different constellations (e.g., share holding companies, co-operatives, foundations) [15]. Due to Sweden´s geography and varying population densities, the educational system is characterised by many small pre-school and school units. For example, in 2017/2018 there were 4832 school units of which circa 83% were public owned and 54% had 199 or fewer students [26]. Typically, publicly owned schools have more students than privately owned schools. While the organization of the schoolwork typically is hierarchically organised around teacher teams, and teacher leaders, the state directives allow for creating locally tailored school units and local organisations. Accordingly, school units may vary as regards the number of school principals, assistant school principals, teachers, and adjacent staff as well as the amount of support and access the school owner provide. Regarding personnel, more females than males are employed as principals, and the proportion of female school principals varies from about 66 to 93% contingent on school level [27, 28]. Official estimates of the mean age of school principals vary from 48 to 52 years [28, 29].

### Participants

Because there is no accessible official register of school principals, participants were recruited via a list of e-mail addresses that covered principals who, during the period 2008–2017, had participated in training programmes funded and arranged by the Swedish National Agency for Education and run by different universities in Sweden. Of the 9900 principals who were invited via e-mail, 4640 potential responders either accepted (*n* = 2633) or declined (*n* = 2007) participation (i.e., a 47% response rate). Of these, 2317 responded, yielding a response rate of 23% in relation to all invited and 50% in relation to those who actively responded to the invitation. The inclusion criteria were met by 2219 school principals who worked at least half time (i.e., 50%) while being employed in a pre-school (28%), pre-school and compulsory school (5%), compulsory school (44%), upper secondary school (15%) or adult education (7%). Their mean age was 49.3 years (SD 7.4 years); 78% were women (*n* = 1724), 22% were men (*n* = 491), and 0.2% (*n* = 4) did not disclose their gender. However, in the present study, only principals and assistant principals who were accountable for zero to 100 subordinates were included. The decision to use an upper limit of 100 subordinates was based on knowledge about the organization of the Swedish educational system and to avoid suspected typographical errors and inaccurate data entries that erroneously inflated estimates of central tendencies and spread (see also description of SoC in measures). Of the 2045 eligible school principals and assistant principals, 77.2% were women (*n* = 1578), 22.6% were men (*n* = 463), and 0.2% (*n* = 4) did not disclose their gender. The mean age in the total group (*N* = 2045) was 49.2 years (SD 7.4 years). For women the mean age was 49.5 years (SD 7.2 years) and for men 48.6 years (SD 7.9 years).

### Measures

#### Span of control

SoC was assessed using the question “*How many employees are you the direct manager of?”.* Responses were given by entering the number of subordinates in a free-text field. We used median and quartile splits to create four subgroups with incrementally wider SoCs: 0 to 22 employees, 23 to 30 employees, 31 to 39 employees, and 40 to 100 employees. This approach was selected in the absence of a gold standard for cut-off values and to enable us to identify a potentially non-linear relationship between SoC and our selected outcomes.

#### Exhaustion symptoms

*The Lund University Checklist for Incipient Exhaustion* (LUCIE) was used to assess exhaustion symptoms of potential relevance to detecting, monitoring, and managing exhaustion disorder (ED) (F43:8 A), as defined in the Swedish version of the International Classification of Diseases 10th Revision, ICD-10-SE [30]. LUCIE comprises 28 items (statements) that cover the following 6 domains: (a) sleep and recovery (3 items), (b) separation between work and spare time (4 items), (c) sense of community and support in the workplace (2 items), (d) managing work duties and personal capabilities (5 items), (e) private life and spare time activities (3 items), and (f) health complaints (11 items) [31, 32]. Responses to the items are given on a 4-point scale: 1 = Not at all, 2 = Somewhat, 3 = Quite a bit, and 4 = Very much. The scoring builds on two algorithms comprising two separate but supplementary scales: the *Stress Warning Scale* (SWS) and the *Exhaustion Warning Scale* (EWS), which are in practice combined into a 4-step ranking of incremental stress symptomatology. The combination of the SWS and EWS scales yields the following four steps: *Step 1-GG* (SWS green zone and EWS green zone), indicating no signs of stress; *Step 2-YG* (SWS yellow zone and EWS green zone), indicating possible or weak signs of stress; *Step 3-RG* (SWS red zone and EWS green zone), indicating mild to moderate lasting stress symptoms, but less severe than ED; *Step 4-RR* (SWS red zone and EWS red zone), indicating severe stress signs and possible ED. The following articles provide more details and information on scoring procedures and adjacent inventories [32, 33], prospective validation [31], and repeated administration [34].

#### Work ability

Current work ability, in comparison to lifetime best, was assessed using Item 1a from the Work Ability Index (WAI) [35, 36], which is also called the Work Ability Score (WAS) [37]. WAS has been considered a valid proxy for work ability in large-scale studies [6, 38, 39]; responses were given on an 11-point Likert-type scale with verbal anchors at the endpoints (0 = Completely unable to work and 10 = Work ability at its best). The item read: “*If you compare your current work ability with your lifetime best*,* what score would you give your current work ability; we assume that your work ability at its best is valued as 10 points*”. Apart from using the score on the WAS as an outcome, for purposes of analysis, the WAS score was also dichotomized into 0 = Poor to good work ability (0–8), and 1 = Excellent work ability (9–10). This cut-off score was selected as it was previously judged to correspond to good or excellent work ability, as assessed using the complete WAI inventory [40], and it was used in our previous study on work ability [7].

#### Demanding and supportive circumstances in the manager job

The 32-item Gothenburg Manager Stress Inventory (GMSI) – Mini, which builds on GMSI [41] was used to assess the participants’ perceived organizational demands and supportive circumstances as a leader. The GMSI-Mini was compiled by selecting the items most highly correlated within each of the 13 dimensions/areas in GMSI (Österberg, K. personal communication). A detailed description of items and Cronbach’s alphas of the scales are presented in Table 1. The overarching question read: “*In relation to the past six months*,* how often have the following occurred in your work as a leader*?”

**Table 1:** Description of the scales in the 32-item Gothenburg Manager Stress Inventory (GMSI)-Mini (*N* = 2045)

Domain and scale names	Description of item content (non-verbatim UK English translation)	Number of items	Cronbach’s α
Demanding managerial circumstances			
Resource deficits	Insufficient possibilities to influence the allocation of resources to the organization. Lacking resources due to the decisions of superiors, politicians or governmental authorities. Not enough resources to cope with peak loads.	3	0.79
Organizational control deficits	Severe difficulties in implementing the decisions from higher levels in the organization. Difficult to follow how decisions are taken in the organization.	2	0.73
Role conflicts	Conflicts between administrative work, organizational development and co-workers. Not enough time for organizational development. Difficulties in finding time to discuss daily activities with colleagues.	3	0.83
Role demands	Demanding responsibilities for (a) performance and quality; (b) personnel; (c) the work environment; and (d) organizational development.	4	0.80
Group dynamics	Problems with feelings of safety and mutual trust within the co-worker group. Feelings of not knowing what is going on in the co-worker group. Co-workers having trouble accepting the common goals of work.	3	0.78
Buffer function	Demands to be a buffer between co-workers and higher levels in the organization. Demands to explain bad or negative decisions that have been taken by superiors. Superiors expect you to understand and be committed to accepting decisions that are bad for you and your organization.	3	0.86
Co-workers	Demands to help co-workers organize and structure their work. Co-workers structure their work inadequately.	2	0.82
Container function	Demands to deal with co-workers’ frustrations with the fact that work is psychologically challenging. Pressured co-workers burden you with their problems.	2	0.87
Supportive managerial circumstances			
Supportive management	Trust that superiors, when needed, will help in solving work environment problems for the co-workers. Experiencing that superiors express a genuine interest in the job and the problems I have as a leader.	2	0.81
Cooperating co-workers	Feelings that co-workers want to take responsibility for their work. Feelings that co-workers have valuable knowledge that makes the manager’s work easier.	2	0.76
Supportive manager colleagues	Access to proper support from fellow school leader colleagues. Proper possibilities to reflect on and discuss organizational issues with fellow school leader colleagues	2	0.90
Supportive private life	Leisure time interests facilitate relaxation from work and associated problems. Leisure time truly provides opportunities to rest and relax from work.	2	0.87
Supportive organizational structures	Clearly defined authority in the work. Clearly defined area of responsibility and task as a leader.	2	0.82

*Demanding circumstances* were assessed in eight dimensions/problem areas (i.e., resource deficits [3 items], organizational control deficits [2 items], role conflicts [3 items], role demands [4 items], group dynamics [3 items], buffer function [3 items], co-workers [2 items], and container function [2 items]). Responses to the items were given on a 5-point scale indicating frequency:1 = Never, almost never, 2 = Rarely, 3 = Sometimes, 4 = Often, and 5 = Always, almost always.

*Supportive circumstances* were assessed in five dimension/areas of support (i.e., supportive management [2 items], co-operation with co-workers [2 items], supportive manager colleagues [2 items], supportive private life [2 items], supportive organizational structures [2 items]). Responses to the items were given on a 5-point scale indicating degree of agreement: 1 = Applies very poorly, 2 = Applies poorly, 3 = Applies to some extent, 4 = Applies well, and 5 = Applies very well.

For both demands and supportive factors, the mean score for each scale was used as the indicator, with higher scores indicating experiencing more demands or more support, respectively.

#### Background and contrast variables

Data on *age*, *gender*, *job title* (two levels: principal versus assistant principal), *school level* (five levels: Pre-school, Pre-school and compulsory school, Compulsory school, Upper secondary education, Adult education), *school owner* (two levels: municipality versus other [i.e., share holding companies, co-operatives, foundations, economic co-operations, non-profit association, county council, individual company etcetera]), and *years of work experience as a school leader* (four levels: up to 3 years, > 3 to 5 years, > 5 to 10 years, > 10 years) were used for demographic description and/or to create internal comparison groups.

### Statistical analysis

The IBM SPSS software (version 29) was used for statistical analysis. We adopted an explanatory modelling approach [42], and two-tailed p-values ≤ 0.05 were considered statistically significant.

For nominal or categorical data, between-group comparisons were conducted using Pearson’s Chi-square test. Kruskal-Wallis H-tests (KW-test) and univariate F-tests were used to examine whether the distribution of continuous SoC scores differed across subgroups defined by gender, job title, school level, school owner, and years of working as a principal.

The GMSI subscale scores were approximately normally distributed. Pearson correlations indicated positive correlations within the two major dimensions demanding managerial circumstances (r’s between 0.15 and 0.60, with most *r* < 0.5) and supportive managerial circumstances (r’s between 0.09 and 0.38). The GLM module and the multivariate procedure were used to perform multiple multivariate analyses of variance (MANOVAs). The independent variable was SoC (4 levels), and the dependent variables were either the eight demanding or the five supportive subscales in the GMSI. Four multivariate outliers were identified for the GMSI demands scales by calculation of Mahalanobis distance within each SoC group, using the linear regression function and comparing the values against a chi-square distribution for eight dependent variables corresponding to a cut-off value of 26.13 at an alpha level of 0.001. Because the outliers represented genuine data and comprised an infinitesimal part of the study sample, they were kept in the analyses. No multivariate outliers were identified for the GMSI support scales.

As SoC differed across job title (two levels), school level (five levels), school owner (two levels), and years of work experience as a school leader (four levels), we also adjusted for these variables in the final analyses. In view of the varying group sizes, we report the Pillai’s Trace multivariate statistic for the MANOVAs. Follow-up analyses were conducted using univariate F-tests and Bonferroni adjusted post-hoc testing for pair-wise differences.

To ascertain the robustness of our MANOVA results and the subsequent follow-up testing, we also performed unadjusted univariate KW-tests (see Table 4  for p-values). Because the KW-tests yielded almost the identical pattern of associations, we chose the parametric method, as this allowed us to calculate estimates and p-values from partially (Model 1) and fully adjusted (Model 2) models. Model 1 comprised adjustment for job title, school level, and years of work experience as a school principal. Model 2 extended Model 1 to also include school owner as an indicator of extra-organizational circumstances.

As some principals had zero (*n* = 30) or just a few subordinates (*n* = 59), we also performed sensitivity analyses by excluding 89 principals who scored below the fifth percentile on the SoC variable. The sensitivity analyses (*n* = 1956) yielded a similar pattern of results and highly similar estimates for both unadjusted and adjusted models (data not shown). For this reason, we settled on presenting the results from the most inclusive model (*N* = 2045).

## Results

The mean SoC in the total study sample was 31.7 employees (SD 15.5), and the median SoC was 30 employees (Table 2).

**Table 2:** Descriptives: Span of Control levels for the total study sample and across gender, job title, school type, and years working as a school leader

Span of Control				Percentiles									Percent	ANOVA F-test	
	^1^N	M	SD	05	25		50	75	95	Min		Max	Women/Men	*P*-value	*p*-η2
Total	2045	31.7	15.5	7	22		30	40	60	0		100	78 / 22		
Gender														0.971	--
^2^Women	1578	31.7	15.7	7	22		30	40	60	0		100	--		
^2^Men	463	31.7	15.1	8	22		30	40	60	0		100	--		
Job title^a^														< 0.001	0.05
Principal	1648	33.3	15.7	8	24		32	40	60	0		100	78 / 22		
Assistant principal	397	24.9	12.8	0	18		25	34	45	0		97	75 / 25		
School level ^b^														< 0.001	0.04
Pre-School	546	33.2	18.0	5	24		32	42	65	2		100	97 / 3		
Pre-school and compulsory school	60	28.7	15.0	5.5	18		29.5	39.5	54.5	0		60	87 / 13		
Compulsory school	963	33.5	14.7	12	24		32	40	60	0		97	73 / 27		
Upper secondary education	332	27.0	12.3	5	20		27.5	35	47	0		70	61 / 39		
Adult education	144	25.8	14.3	4	15		25.5	35	46	0		80	65 / 35		
Owner ^c^														< 0.001	0.11
Municipality	1605	34.3	14.4	12	25		33	40	60	0		100	77/23		
Other	440	22.0	15.5	3	10.5		20	30	50	0		95	77/23		
Years as school leader ^d, e^														< 0.001	0.02
up to 3 years	393	28.2	14.4	4	20		28	36	52	0		97	84 / 16		
> 3 to 5 years	463	31.1	14.8	8	23		30	40	60	0		95	80 / 20		
> 5 to 10 years	711	32.8	15.6	9	23		32	40	60	0		100	77 / 23		
> 10 years	478	33.5	16.5	7	24		33	41	65	2		100	70 / 30		

*M* = Mean, *SD* = Standard deviation, p-η2 = Partial eta square

^1^Four individuals did not disclose their gender. ^2^The almost identical estimates for men and women are correct

a = includes the job titles pre-school director (*n* = 556) and principal and pre-school director (*n* = 68), which from July 1 2019 were renamed “principal”

b = post-hoc testing indicates that the sample distributions are significantly different at *p* < 0.05 (Bonferroni adjusted) for the following pair-wise tests: Adult education vs. Pre-School; Adult education vs. Compulsory school; Upper secondary education vs. Pre-School; Upper secondary education vs. Compulsory school

c = The other category comprises several types of school owners including share holding companies, cooperatives, foundations, etc

d = post-hoc testing indicates that the sample distributions are significantly different at *p* < 0.05 (Bonferroni adjusted) for the following pair-wise tests: Up to 3 years vs. >5 to 10 years; Up to 3 years vs. > 10 years

e = The outermost categories (i.e., < 1 year (*n* = 12), and > 20 years (*n* = 89) are merged with adjacent category

### Span of control across groups defined by demographic and organizational factors

Male and female school principals reported similar levels of SoC. However, differences in SoC scores were observed regarding the school principal’s job title, school level, school owner, and the years they had worked as a school leader (Table 2). Assistant principals, principals working in adult education, and principals who had worked less than three years as a school principal had the narrowest spans of control. Principals in schools that were owned by municipalities had on average a broader SoC compared with principals working for other organizations that own and operate schools (Table 2). Post-hoc testing further indicated differences between principals who worked in adult education when compared with principals working in pre-school and compulsory school. Principals working in upper-secondary school had a lower SoC than principals working in pre-schools and compulsory school. Furthermore, principals who had worked less than three years had a lower SoC than principals who had worked more than three years (Table 2).

### Span of control across exhaustion and work ability groups

The distribution of SoC scores across the four LUCIE ranking steps were not significantly different (χ2 = 9.39, df = 9; *p* = 0.402), indicating an even distribution across SoC levels as regards reports of stress and exhaustion (Table 3).

The prevalence proportion for excellent work ability (i.e., reporting 10 or 9 on WAS) was 29.7% (*n* = 608), and for poor to moderate work ability (i.e., reporting 8 or below on WAS) it was 70.3% (*n* = 1437). The distribution of SoC scores across the two work ability groups was not significantly different (χ2 = 3.53, df = 3; *p* = 0.317), indicating an even distribution across SoC scores regarding ratings of work ability (Table 3).

**Table 3:** Distribution of LUCIE and Work ability scores across levels of Span of Control. (*N* = 2045)

	Span of Control								
	0–22 employees		23–30 employees		31–39 employees		40–100 employees		
	(*n* = 516)		(*n* = 517)		(*n* = 475)		(*n* = 537)		χ2-test
	**N**	**%**	**N**	**%**	**N**	**%**	**N**	**%**	P**-value**
LUCIE^1^									0.402
Step 1-GG	267	52.0	257	49.7	223	47.0	259	48.5	
Step 2-YG	119	23.2	139	26.9	132	27.8	140	26.2	
Step 3-RG	76	14.8	63	12.2	77	16.2	87	16.3	
^ 2^Step 4-RR	51	9.9	58	11.2	42	8.9	48	9.0	
Workability score^3^									0.317
Poor to good work ability	444	86.0	464	89.7	413	86.9	470	87.5	
Excellent work ability	72	14.0	53	10.3	62	13.1	67	12.5	

^1^ Step 1-GG (SWS green zone and EWS green zone), indicating no signs of stress; Step 2-YG (SWS yellow zone and EWS green zone), indicating possible, or weak signs, of stress; Step 3-RG (SWS red zone and EWS green zone), indicating clear signs of stress but not of exhaustion; Step 4-RR (SWS red zone and EWS red zone), indicating severe stress signs and possible ED

^2^The rare combination of SWS yellow + UWS red was included in LUCIE category Step 4-RR (*n* = 7)

^3^Poor to good work ability = 0–8; Excellent work ability = 9–10

### Span of control and demanding managerial circumstances

For the eight demanding managerial circumstances in GMSI, the unadjusted multivariate analysis (MANOVA) indicated a main effect difference across levels of SoC (Main effect: Pillai-Bartlett trace V = 0.074, F = 6.41, *p* < 0.001, partial eta squared = 0.03).

Univariate follow-up F-tests indicated differences across the four levels of SoC on seven of the eight GMSI scales: resource deficits, organizational control deficits, role conflicts, role demands, buffer function, co-workers, and container function (Table 4). With one exception, the bivariate post-hoc testing showed that a control span between zero and 22 employees was associated with less frequent experiences of demanding managerial circumstances as compared with the other control spans (i.e., 23 to 30 employees; 31 to 39 employees; and 40 to 100 employees) (Table 4). Non-parametric KW-tests confirmed the robustness of this observed pattern of association (Table 4).

**Table 4:** Bonferroni adjusted univariate follow-up F-tests: Unadjusted estimated marginal means and accompanying 95% Confidence Intervals (95% CI) for demanding and supportive managerial circumstances across levels of span of control (*N* = 2045)

	Span of Control								Univariate tests		
	0–22 employees **( = 516)**		23–30 employees **(n = 517)**		31–39 employees **(n = 475)**		40–100 employees **( n = 537)**		F-test		KW-test
**Domain and scale names**	**Mean**	**95% CI**	**Mean**	**95% CI**	**Mean**	**95% CI**	**Mean**	**95% CI**	**P -value**	**p-η2**	**P -value**
*Demanding managerial circumstances*											
Resource deficits	3.22 _a_	(3.14–3.31)	3.47 _b_	(3.38–3.55)	3.52 _b_	(3.44–3.61)	3.48 _b_	(3.40–3.56)	< 0.001	0.02	< 0.001
Organizational control deficits	2.36 _a_	(2.28–2.43)	2.60 _b_	(2.52–2.67)	2.64 _b_	(2.56–2.71)	2.56 _b_	(2.49–2.64)	< 0.001	0.02	< 0.001
Role conflicts	3.42 _a_	(3.35–3.49)	3.72 _b_	(3.65–3.79)	3.85 _b, c_	(3.77–3.92)	3.88 _c_	(3.81–3.95)	< 0.001	0.05	< 0.001
Role demands	2.92 _a_	(2.85–2.98)	3.08 _b_	(3.01–3.15)	3.12 _b_	(3.05–3.20)	3.19 _b_	(3.12–3.26)	< 0.001	0.02	< 0.001
Group dynamics	2.30	(2.23–2.36)	2.42	(2.35–2.48)	2.35	(2.28–2.42)	2.39	(2.33–2.46)	0.057	--	0.034
Buffer function	2.77 _a_	(2.69–2.85)	2.98 _b_	(2.90–3.05)	2.98 _b_	(2.90–3.06)	2.90 _b_	(2.83–2.98)	< 0.001	0.01	< 0.001
Co-workers	2.90	(2.83–2.96)	2.86	(2.80–2.92)	2.92	(2.85–2.98)	2.93	(2.87–3.00)	0.399	--	0.377
Container function	3.11 _a_	(3.04–3.19)	3.35 _b_	(3.28–3.42)	3.43 _b_	(3.36–3.51)	3.45 _b_	(3.38–3.52)	< 0.001	0.03	< 0.001
*Supportive managerial circumstances *											
Supportive management	3.39 _a_	(3.29–3.48)	3.09 _b_	(3.00–3.19)	3.19 _b_	(3.10–3.29)	3.17 _b_	(3.08–3.26)	< 0.001	0.01	< 0.001
Cooperating co-workers	4.18	(4.13–4.24)	4.22	(4.16–4.28)	4.18	(4.12–4.24)	4.22	(4.16–4.27)	0.694	--	0.739
Supportive manager colleagues	3.62 _a_	(3.53–3.71)	4.02 _b_	(3.93–4.11)	4.01 _b_	(3.92–4.11)	3.92 _b_	(3.83–4.01)	< 0.001	0.02	< 0.001
Supportive private life	3.83	(3.74–3.92)	3.78	(3.69–3.86)	3.85	(3.75–3.94)	3.85	(3.76–3.93)	0.641	--	0.777
Supportive organizational structures	3.60 _a_	(3.52–3.68)	3.67 _a_	(3.59–3.75)	3.58 _a_	(3.50–3.67)	3.73 _a_	(3.65–3.81)	0.036	0.00	0.070

Values in the same row and sub-table not sharing the same subscript are significantly different at *p* < 0.05

p-η2 = partial eta square

KW = Kruskal-Wallis H-test

Adjusting for job title, school level, and years of working as a principal did not alter the pattern of results. Hence, the MANOVA indicated a main effect difference across SoC groups (Main effect: Pillai-Bartlett trace V = 0.069, F = 6.01, *p* < 0.001, partial eta squared = 0.02). Moreover, univariate follow-up F-tests indicated differences across the four levels of SoC on seven of the eight GMSI scales: resource deficits, organizational deficits, role conflicts, role demands, group dynamics, buffer function, co-workers, and container function (Table 5). With one exception, the bivariate post-hoc testing showed consistently that principals with an SoC between zero and 22 employees reported less frequent experiences of demanding managerial circumstances as compared with the three control spans (Table 5).

**Table 5:** Bonferroni adjusted univariate follow-up F-tests: Estimated marginal means and accompanying 95% Confidence Intervals (95% CI) for demanding and supportive managerial circumstances across levels of span of control (*N* = 2045). Model 1: adjusted for job title, school level and years as a school leader

	Span of Control								Univariate tests	
	0–22 employees **(n = 516)**		23–30 employees **( n = 517)**		31–39 employees **( n = 475)**		40–100 employees **( n = 537)**		F-test	
**Domain and scale names**	**Mean**	**95% CI**	**Mean**	**95% CI**	**Mean**	**95% CI**	**Mean**	**95% CI**	**P -value**	**p-η2**
*Demanding managerial circumstances*										
Resource deficits	3.14 _a_	(3.04–3.24)	3.36 _b_	(3.26–3.46)	3.40 _b_	(3.29–3.51)	3.35 _b_	(3.24–3.46)	< 0.001	0.01
Organizational control deficits	2.36 _a_	(2.27–2.45)	2.62 _b_	(2.52–2.71)	2.67 _b_	(2.56–2.76)	2.61 _b_	(2.51–2.71)	< 0.001	0.02
Role conflicts	3.35 _a_	(3.27–3.44)	3.65 _b_	(3.56–3.74)	3.77 _b, c_	(3.68–3.87)	3.81 _c_	(3.72–3.91)	< 0.001	0.04
Role demands	2.87 _a_	(2.79–2.95)	3.03 _b_	(2.95–3.12)	3.07 _b_	(2.98–3.16)	3.15 _b_	(3.06–3.24)	< 0.001	0.02
Group dynamics	2.34 _a_	(2.27–2.42)	2.49 _b_	(2.41–2.57)	2.45 _a, b_	(2.36–2.53)	2.52 _b, c_	(2.44–2.61)	< 0.001	0.01
Buffer function	2.74 _a_	(2.65–2.84)	2.96 _b_	(2.86–3.06)	2.97 _b_	(2.86–3.08)	2.91 _b_	(2.80–3.01)	< 0.001	0.01
Co-workers	2.91	(2.83–2.98)	2.88	(2.80–2.95)	2.93	(2.85–3.02)	2.95	(2.87–3.03)	0.408	--
Container function	3.11 _a_	(3.03–3.19)	3.35 _b_	(3.26–3.44)	3.44 _b_	(3.34–3.53)	3.47 _b_	(3.37–3.56)	< 0.001	0.02
*Supportive managerial circumstances*										
Supportive management	3.44 _a_	(3.33–3.55)	3.16 _b_	(3.05–3.28)	3.28 _b_	(3.15–3.40)	3.26 _b_	(3.14–3.39)	< 0.001	0.01
Cooperating co-workers	4.15	(4.08–4.21)	4.16	(4.09–4.23)	4.11	(4.04–4.19)	4.13	(4.05–4.21)	0.740	--
Supportive manager colleagues	3.64 _a_	(3.54–3.75)	4.04 _b_	(3.93–4.16)	4.07 _b_	(3.94–4.19)	3.98 _b_	(3.86–4.10)	< 0.001	0.03
Supportive private life	3.87	(3.76–3.97)	3.82	(3.71–3.93)	3.89	(3.77–4.00)	3.88	(3.76–4.00)	0.686	--
Supportive organizational structures	3.54	(3.45–3.63)	3.58	(3.48–3.68)	3.48	(3.38–3.58)	3.59	(3.48–3.69)	0.230	--

Values in the same row and sub-table not sharing the same subscript are significantly different at *p* < 0.05

p-η2 = partial eta square (unique contribution in relation to the total model)

Adjusting for school owner, however, altered the pattern of results. While the MANOVA indicated a main effect difference between SoC groups (Main effect: Pillai-Bartlett trace V = 0.040, F = 3.40, *p* < 0.001, partial eta squared = 0.01), the univariate follow-up F-tests indicated only differences on three of the eight GMSI scales (i.e., role conflicts, role demands, and container function) (Table 6). Bivariate post-hoc testing showed that principals with an SoC between zero and 22 employees reported less frequent role demands and that they less frequently needed to harbour the employees’ frustration (i.e., container function) as compared with principals with broader SoCs (Table 6). Compared with all other groups, principals with an SoC between zero and 22 also reported less frequent role conflicts. Moreover, principals with a control span of 23–30 employees reported less frequent role conflicts than principals with the broadest SoC (i.e., 40–100), but did not differ from principals with an SoC of 31–39 employees (Table 6).

**Table 6:** Bonferroni adjusted univariate follow-up F-tests: Estimated marginal means and accompanying 95% Confidence Intervals (95% CI) for demanding and supportive managerial circumstances. Model 2: adjusted for job title, school level, years as a school leader and school owner

	Span of Control								Univariate tests	
	0–22 employees (*n* = 516)		23–30 employees (*n* = 517)		31–39 employees (*n* = 475)		40–100 employees (*n* = 537)		F-test	
Domain and scale names	Mean	95% CI	Mean	95% CI	Mean	95% CI	Mean	95% CI	*P*-value	*p*-η2
*Demanding managerial circumstances*										
^ a^ Resource deficits	3.15	(3.06–3.25)	3.20	(3.09–3.30)	3.20	(3.09–3.32)	3.16	(3.04–3.28)	0.786	--
^ b^ Organizational control deficits	2.38	(2.29–2.46)	2.40	(2.31–2.50)	2.40	(2.30–2.51)	2.37	(2.26–2.47)	0.858	--
^ c^ Role conflicts	3.36 _a_	(3.28–3.45)	3.52 _b_	(3.43–3.61)	3.62 _b, c_	(3.52–3.72)	3.67 _c_	(3.57–3.77)	< 0.001	0.02
^ d^ Role demands	2.87 _a_	(2.79–2.95)	3.01 _b_	(2.92–3.10)	3.05 _b_	(2.95–3.14)	3.12 _b_	(3.03–3.22)	< 0.001	0.01
^ e^ Group dynamics	2.35	(2.28–2.42)	2.39	(2.31–2.47)	2.32	(2.23–2.41)	2.41	(2.32–2.49)	0.273	--
^ f^ Buffer function	2.75	(2.66–2.85)	2.82	(2.72–2.92)	2.80	(2.69–2.91)	2.75	(2.63–2.86)	0.478	--
^ g^ Co-workers	2.91	(2.83–2.98)	2.88	(2.80–2.96)	2.94	(2.85–3.03)	2.95	(2.87–3.04)	0.390	--
^ h^ Container function	3.12 _a_	(3.03–3.20)	3.27 _b_	(3.18–3.37)	3.35 _b_	(3.25–3.45)	3.38 _b_	(3.28–3.49)	< 0.001	0.01
*Supportive managerial circumstances*										
^ i^ Supportive management	3.43 _a_	(3.33–3.54)	3.25 _b, c_	(3.13–3.37)	3.38 _a, c_	(3.25–3.51)	3.36 _a, c_	(3.23–3.50)	0.049	0.00
^ j^ Cooperating co-workers	4.14	(4.08–4.21)	4.18	(4.10–4.25)	4.14	(4.06–4.22)	4.16	(4.07–4.24)	0.780	--
^ k^ Supportive manager colleagues	3.65 _a_	(3.54–3.75)	3.94 _b_	(3.82–4.06)	3.94 _b_	(3.82–4.07)	3.87 _b_	(3.74–4.00)	< 0.001	0.01
^ l^ Supportive private life	3.87	(3.77–3.97)	3.78	(3.67–3.90)	3.85	(3.72–3.97)	3.84	(3.72–3.97)	0.581	--
^ m^ Supportive organizational structures	3.54	(3.45–3.63)	3.58	(3.48–3.69)	3.48	(3.37–3.59)	3.59	(3.48–3.70)	0.230	--

Values in the same row and sub-table not sharing the same subscript are significantly different at *p* < 0.05;

p-η2 = partial eta square (i.e., the unique proportion of the variance the independent variable explains in relation to the dependent variable [i.e., span of control] when adjusted for Job title, School level, Owner, and years as a school leader)

The R Squared values for the models that assessed each demanding managerial circumstances predictor (from a to h) were as follows from top to bottom: ^a^ R Squared = 0.07; ^b^ R Squared = 0.11; ^c^ R Squared = 0.10; ^d^ R Squared = 0.04; ^e^ R Squared = 0.06; ^f^ R Squared = 0.05; ^g^ R Squared = 0.05; ^h^ R Squared = 0.07. The R Squared values for the models that assessed each supportive managerial circumstances predictor (from i to m) were as follows from top to bottom: ^i^ R Squared = 0.03; ^j^ R Squared = 0.05; ^k^ R Squared = 0.05; ^l^ R Squared = 0.01; ^m^ R Squared = 0.04

### Span of control and supportive managerial circumstances

For the five scales assessing supportive managerial circumstances in GMSI,

the unadjusted multivariate analysis (MANOVA) indicated main effect differences across levels of SoC (Main effect: Pillai-Bartlett trace V = 0.057, F = 7.89, *p* < 0.001, partial eta squared = 0.02).

Univariate follow-up tests indicated differences across the four levels of SoC in three of the five supportive organizational factors, as assessed by the GMSI: supportive management, supportive manager colleagues, and supportive organizational structures (Table 4). Yet bivariate post-hoc testing indicated only pairwise differences for supportive management and supportive manager colleagues. While the principals with the lowest SoC reported more support from managers, they reported less support from leader colleagues (Table 4).

Adjusting for job title, school level, and years of working as a principal did not alter the pattern of results. Thus, the MANOVA indicated a main effect difference between SoC groups and the GMSI scales (Main effect: Pillai-Bartlett trace V = 0.055, F = 7.52, *p* < 0.001, partial eta squared = 0.02), and univariate follow-up F-tests, as well as subsequent bivariate post-hoc testing, showed that principals with the lowest SoC reported more support from managers but less support from manager colleagues (Table 5).

Adjusting for school owner did not alter the pattern of results. Hence, the MANOVA indicated a main effect difference between SoC groups and the GMSI scales (Main effect: Pillai-Bartlett trace V = 0.027, F = 3.68, *p* < 0.001, partial eta squared = 0.01).

Univariate follow-up F-tests, and subsequent bivariate post-hoc testing, showed that principals with the lowest SoC reported more support from managers but less support from leader colleagues (Table 6).

## Discussion

The present study assessed SoC among Swedish school principals working in both public and independent pre-schools, compulsory schools, upper secondary schools, and adult education. Subsequently, we examined how SoC was associated with the occurrence of exhaustion symptoms, perceived work ability, as well as demanding and supportive managerial circumstances.

The results showed that the mean SoC was 31.7 employees (SD = 15.5; median 30). No difference was observed between male and female school principals. Even if caution should be exercised when making comparisons across organizations and studies, our results differ from a study including 434 Swedish public sector managers, which reported that the average SoC was 27.4 employees (SD = 14.99) and that men had a lower SoC than women (23 and 29 subordinates, respectively) [22]. At first glance, the fact that compulsory school principals on average were accountable for 33.5 employees (median 32 employees) seems to differ from the Swedish National Agency for Education estimates, which state that compulsory school principals on average are accountable for 28 teachers [11]. However, because our definition of SoC comprises all employees the school principal is accountable for, not just teachers, it cannot be excluded that the differences are smaller or even absent.

Nonetheless, and within our study sample, SoC differed across groups of school principals defined by job title, school level, school owner, and years of working as a school principal. As expected, considering the implied work roles, school principals reported a wider SoC than assistant principals (median 32 and median 25, respectively). It was also more common for school principals and assistant principals working in upper secondary school and adult education to report a narrower SoC than school principals and assistant principals working in pre-schools or compulsory schools. That school principals working in municipality owned schools reported having a broader SoC than school principals working in independent schools was expected. However, the large difference in median SoC (i.e., 33 Vs. 20, respectively) was a bit surprising and is probably in part attributable to school owners deliberate strategies and unique pre-conditions as well as the fact that our assessment of independent schools (i.e., other) comprised many types of school owners (e.g., individual companies and co-operatives). For this reason, conclusions concerning independent schools should be made with caution. In addition, school principals and assistant principals with experience of working as a principal less than three years reported on average a narrower SoC than school principals and assistant principals who had worked as a school principal for more than five years. To what extent the reports of narrower or broader SoCs in certain school levels, or among less experienced principals, is contingent on a deliberate organization of work or merely reflects unspoken traditions, norms, or external circumstances (e.g., a smaller number of students in non-mandatory school levels) is not known and cannot readily be assessed using our data. However, from the perspective that the educational system is governed by the state via statutes, rules, national curriculums, and syllabuses, and that it is up to the municipalities, independent school owners, and local school units to organize the education [15], it is clear that decisions concerning SoC can be made on various levels (e.g., on the national or school unit level) as well as by different means (e.g., legislation versus self-regulation).

The lack of an association between SoC and early and manifest exhaustion-related symptoms in LUCIE indicates that the number of employees a school principal is accountable for is not directly linked to the principal’s mental health. Likewise, the absence of an association between SoC and self-rated current work ability compared to the lifetime best indicates that SoC does not directly affect the principal’s own judgments of performance capacity. Importantly, however, this does not exclude that SoC may be a contributing factor to situations and circumstances that in the long run may impact school principals’ mental health and/or work ability. Noticeably, SoC constitutes an organizational precondition that influences the time a manager has to think about individual employees as well as to interact and build relationships with them, which may impact behaviours such as co-ordination, feedback, and support. From this perspective, it is interesting to note that the mean scores, and the absence of associations between SoC scores and GMSI scale scores for the “co-workers” and “co-operating co-workers” scales, paint a generally positive picture and suggest that the nature and quality of the interactions between school principals and their employees are good and likely to facilitate effective management. Indeed, the school principals report that they seldom need to help their employees organize and structure their work and that they feel their employees take responsibility for their work and have valuable knowledge that makes their own executive work esier. The results also suggest that, compared with school principals with broader SoCs, school principals with the narrowest SoC have greater trust that their superiors will help them and perceive that their superiors have a genuine interest in their work. However, the school principals with the narrowest SoC also perceive that they have less access to support from manager colleagues and thus fewer possibilities to discuss organizational issues with them.

Similar to Wallin and colleagues, who examined SoC among public sector managers, and who observed associations with five of the seven demands studied [22], our unadjusted statistical analyses showed associations with seven of the eight demand scales in the GMSI. However, while Wallin and colleagues identified excessive role demands and demands to deal with co-workers’ frustrations and problems (i.e., a container function) as the managerial circumstances most strongly associated with SoC [22], we found role conflicts (e.g., conflicts between administrative work, not enough time for organizational development or to discuss daily activities with colleagues) to be the factor most strongly associated with SoC in both the unadjusted and adjusted models. Although the partial eta-square estimates from the unadjusted and adjusted univariate models are numerically small, they do suggest that SoC makes a small but consistent and unique contribution to GMSI scale scores. Also, in light of relationship-based approaches to leadership that emphasize that both the nature and quality of interaction between managers and employees are important for effective leadership [17], it seems conceivable that the strength of associations between SoC and GMSI scores (as well as the scores from the other variables here measured) is contingent on the seemingly good relationships that exists between school principals and their employees in the present study sample. In extension, this suggest that future research might benefit from examining the inherently quantitative SoC construct in the light of more qualitative constructs that may help capture the underlying nature of the relationship between managers and employees such as trust, mutual respect and/or even variation in individual motivations.

In contrast to Wallin and colleagues [22], who adopted a linear approach to SoC, and Jacobsen and colleagues who found that a medium SoC (i.e., 30 to 40 individuals) was better than a narrower (i.e., 6 to 29 individuals) or broader (i.e., 41 to 118 individuals) one in a sample of 393 managers and 1699 nurses from Danish hospitals [18], our results seem to be more in line with Cathcart and colleagues’ studies of hospital employees [19], as they suggest the existence of a “critical point” or a threshold effect. Indeed, it was an almost consistent finding that the group of school principals with the narrowest SoC (zero to 22 employees) provided the most desirable GMSI results. Noticeably, our sensitivity analyses that excluded principals reporting an SoC between zero and six (i.e., defining the narrow group to be between 7 and 22 employees) confirmed the robustness of the results. Moreover, because research has failed to identify specific numbers, or cut-off points, for SoC, and in line with previous studies [18], we used median and quartile scores to create groups with varying spans of control. While this method is justified in the absence of a gold standard, it is a heuristic that introduces a degree of relativism, as the cut-off points will be sample specific. Hence, it should be acknowledged that the limits used here (i.e., ≤ 22 employees, 23–30 employees, 31–39 employees, and 40–100 employees) point in a direction without providing a definitive answer to the question of how many employees a school principal should have.

At any rate, adjusting for school owner (i.e., municipality vs. other owner forms) reduced the strength of the association between SoC and support from manager colleagues, as well as removing the associations between SoC and the four GMSI variables resource deficits, organizational control deficits, group dynamics, and buffer function. This impact is not particularly surprising, as schools organized and owned by actors other than municipalities are often smaller and operate by design with narrower SoCs. Still, the remaining associations between SoC, on the one hand, and role conflicts, role demands, container function as well as supportive management and supportive manager colleagues, on the other, stand out as robust and indicate that these associations are independent of school owner. It is not known whether deliberate manipulation of SoC could be used to decrease school principals’ experiences of having too little time to discuss daily work-related activities with colleagues while experiencing conflicts between administrative work, organization, and co-workers (i.e., role conflicts), or the excessive demands they experience in relation to performance, staff, work environment, and organizational development (i.e., role demands), or to alleviate their reported need to harbour frustrations expressed by co-workers (i.e., container function). Yet deliberate manipulation of SoC in the health care sector by increasing the number of managers and narrowing the SoC has purportedly been shown to improve employee engagement [19]. The effects on the managers, however, were not disclosed.

### Methodological considerations

Because all data were obtained using self-reports in a cross-sectional study design, common method bias in the form of interrater effects cannot be excluded [43]. While it is reasonable to view SoC as an organizational precondition determined by administrative decisions that are independent of the principal’s mental health, work ability, and perceptions of demanding and supportive circumstances, reverse causality cannot be excluded. For example, it is conceivable that some school principals might have narrower spans of control due to organizational actions and administrative decisions that have been taken to, for example, reduce high workloads or facilitate recovery by limiting the number of employees the principal is accountable for. However, irrespective of the underlying processes that have led to the manifestation of various SoCs, the present cross-sectional study allows conclusions to be drawn concerning associations between SoC and exhaustion symptoms, work ability, and perceived managerial circumstances at the time of investigation.

The external validity of the results is strengthened by the fact that the school principals were recruited on a national basis and from all school levels. Despite varying definitions of school principals in official statistics, it is also a strength that the present study sample, when compared with data from official sources, appears to be reasonably representative of Swedish principals as a group regarding mean age and the distribution of male and female principals across school levels [27, 28]Furthermore, both the internal and external validity of the results are strengthened by using previously tested and validated items such as the WAS score [6] and instruments such as GMSI-mini [7, 41] and LUCIE [31, 32], whose items have been derived from interviews with manager and patient groups, respectively. Other factors that improve the internal validity are the large sample size and the fact that we studied a reasonably homogenous occupational group that can be expected to have similar work tasks, which is a known factor that may influence the SoC in the organization [21]. Indeed, the work of school principals is governed by laws, national curriculums and syllabuses, hierarchically organized, and principals have a shared mission to educate and prepare children and students for life in society [13–15]. However, this does not exclude local variations in administration, school ownership, and student characteristics that may affect how management is carried out and whether being accountable for few or many employees matters.

## Conclusions

The median SoC (i.e., the number of employees the principal is accountable for) among Swedish school principals is estimated to 30 employees, but varies across job title, school level, years as a school leader and school owner. There was no difference between men and women, and SoC was not directly associated with reporting of early or manifest exhaustion symptoms or with perceived work ability. The GMSI ratings show that the school principals on average value their employees and view them as resourceful contributors to the organization, but also that SoC seems to matter for their possibilities to manage. Irrespective of job title, and when compared with other SoC groups, school principals with an SoC of 22 employees or fewer consistently report less demanding organizational managerial circumstances. Especially role conflicts, role demands, demands to harbour employees’ frustrations (i.e., container function) and perceptions of having lesser access to support from manager colleagues appear to be robustly associated with SoC. While not likely to directly affect exhaustion symptoms or perceived work ability, or to improve school principals and assistant principals’ access to supportive manager colleagues, narrowing school principals’ and assistant principals’ SoC by moving towards 22 employees’ or fewer could potentially alleviate the experienced burden of demanding role conflicts, role demands, and having to function as a container for employees’ problems.

## Data Availability

Consistent with the study protocols approved by the Regional Ethical Review Board, and the Swedish Ethical Review Authority, anonymized data are stored locally at Lund University, Lund, Sweden. In accordance with the ethical approval, crude data are not to be published on the internet. Access to data will be granted to eligible researchers wishing to audit our research. Requests should be directed to the corresponding author.
